# Everyday Needs Assessment for Cognitive Tasks: Challenges for Persons With Cognitive Impairment

**DOI:** 10.1093/geroni/igab046.734

**Published:** 2021-12-17

**Authors:** Wendy Rogers, Raksha Mudar, Maurita Harris, Elizabeth Lydon, Widya Ramadhani, Sara Czaja, Walter Boot, Neil Charness

**Affiliations:** 1 University of Illinois Urbana-Champaign, Champaign, Illinois, United States; 2 University of Illinois-Urbana Champaign, Champaign, Illinois, United States; 3 University of Illinois at Urbana Champaign, Champaign, Illinois, United States; 4 Weill Cornell Medicine/Center on Aging and Behavioral Research, New York, New York, United States; 5 Florida State University, Tallahassee, Florida, United States

## Abstract

ENACT (Everyday Needs Assessment for Cognitive Tasks) is an exploration and discovery project to gather information on challenges in daily and community living experienced by individuals aging with compromised cognition due to mild cognitive impairment, traumatic brain injury, or post-stroke. We are exploring their challenges through a longitudinal needs assessment study involving interviews with older adults with cognitive impairment and their care partners. We will describe the study development process wherein we interviewed subject matter experts, including persons with professional (neurology, rehabilitation, gerontology) or personal experience with individuals who have cognitive impairment. Based on their collective insights, we selected the following categories of activities for the ENACT in-depth interviews: health, social engagement, transportation, domestic life, and leisure/recreation. The ENACT longitudinal data will provide insights to guide development of adaptive, context-sensitive technology-based supports for the AUGMENT, DREAM, and STRUMM projects described in this symposium, as well as other initiatives.

